# Supplementary material for: Cross-platform metabolomics imputation using importance-weighted autoencoders
Source: NPJ Syst Biol Appl. 2026 Jan 10;12:23. doi: 10.1038/s41540-025-00644-5 (PMC12894875; doi:10.1038/s41540-025-00644-5)
Supplement: Supplementary file 1 — Supplementary [file 41540_2025_644_MOESM1_ESM.pdf]

Supplementary

Supplementary Data 1. The list of Metabolon metabolites and NPC LC-MS features assigned to each cluster.

Supplementary Data 2. Hyperparameters of the imputation models and their performance on the training and test sets.

Supplementary Data 3. Imputation performance of all Metabolon metabolites in the full and individual cluster models based on held-out test set samples.

Supplementary Data 4. Association results for the imputed and real valued Metabolon metabolites with CRP.

Supplementary Data 5. Association results for the imputed and real valued Metabolon metabolites with BMI.
